# Low-grade endometrial stromal sarcoma: a case report and literature review

**DOI:** 10.3389/fonc.2025.1652010

**Published:** 2025-08-25

**Authors:** Xiaojing Zhao, Chuqiao Chen, Tingting Xie, Liang Wang

**Affiliations:** ^1^ Second Affiliated Hospital, School of Medicine, Zhejiang University, Hangzhou, China; ^2^ Shulan Hangzhou Hospital Affiliated to Zhejiang Shuren University Shulan International Medical College, Hangzhou, China

**Keywords:** case report, low-grade endometrial stromal sarcoma, laparoscopic power morcellation, magnetic resonance imaging, uterine artery embolization

## Abstract

**Background:**

Low-grade endometrial stromal sarcoma (LG-ESS) is a rare malignant tumor of the female reproductive system with atypical clinical symptoms and slow progression.

**Case:**

A 44-year-old female with a history of intermittent severe dysmenorrhea, previous laparoscopic myomectomy, and uterine artery embolization (UAE) presented with rapidly enlarging pelvic masses. Imaging revealed uterine masses suggestive of leiomyomas, although an adnexal origin could not be excluded. Laparoscopy showed an irregularly enlarged uterus with widespread pelvic masses. Postoperative pathology and immunohistochemistry confirmed a diagnosis of LG-ESS with focal sex cord-like and smooth muscle differentiation. The patient underwent total hysterectomy and bilateral salpingo-oophorectomy, with subsequent recommendations for aromatase inhibitor therapy and lifelong follow-up. The 3-month post-surgery follow-up results indicated that the patient was recovering well, with no signs of recurrence.

**Conclusion:**

By reviewing the patient’s medical history, we gained new insights into the etiology, risk factors, and imaging characteristics of LG-ESS. Endometriotic lesions may serve as a source of LG-ESS. The use of morcellators and UAE in the treatment of benign uterine conditions can increase the risk of malignant tumors. Distinguishing LG-ESS on imaging can be challenging, but magnetic resonance imaging shows some suggestive features, including isointense or slightly hypointense signals on T1-weighted imaging, a mixed signal on T2-weighted imaging or a homogeneous high signal with internal hypointense bands, a high signal on diffusion-weighted imaging, and low apparent diffusion coefficient values, which may aid in differentiation.

## Introduction

Endometrial stromal tumors (ESTs) are rare tumors originating from endometrial stromal cells. ESTs are classified into four types by the World Health Organization (WHO) 2020 classification: endometrial stromal nodule (ESN), low-grade endometrial stromal sarcoma (LG-ESS), high-grade endometrial stromal sarcoma (HG-ESS), and undifferentiated uterine sarcoma (UUS). ESN is a benign tumor, while the other three are collectively referred to as endometrial stromal sarcomas (ESSs). Despite making up less than 1% of malignant uterine tumors, ESSs are the second most common uterine sarcoma after uterine leiomyosarcoma, accounting for 17-25% of cases (1). The most common type of ESSs, known as LG-ESS, is a low-grade malignant tumor with slow progression and late recurrence. LG-ESS primarily affects perimenopausal women between the ages of 40 and 55, with onset ranging from 11 to 76 years (2, 3).

Patients commonly present with non-specific symptoms such as abnormal uterine bleeding, dysmenorrhea, and pelvic pain; however, around 25% remain asymptomatic. According to research, obesity, diabetes, early menarche, long-term tamoxifen or estrogen supplementation, and a history of pelvic irradiation have all been associated with an increased risk of developing LG-ESS (1, 4–6).

Due to its mild symptoms, low morbidity, and lack of specific imaging features, LG-ESS is often misdiagnosed as uterine leiomyoma, adenomyoma, or malignant tumor elsewhere. The International Federation of Gynecology and Obstetrics (FIGO) stage is considered the principal prognostic indicator in LG-ESS. About 60% of LG-ESS cases are identified at FIGO stage I, whereas merely 20% are detected at stage IV metastatic disease (7). Research demonstrated that individuals with FIGO stage ≥ II were approximately 6.6 times more likely to experience recurrence, mortality, or refractory disease than those with stage I (8); the five-year survival rate for patients with stages I and II exceeded 90%, whereas it decreased to 50% for those with stages III and IV (9).

Here, we share a case of stage IIIB LG-ESS in a patient who was misdiagnosed with multiple uterine leiomyomas during long-term follow-up. Written consent has been obtained from the patient, and the case report has received approval from the local ethics committee.

## Case

**Figure 1:**
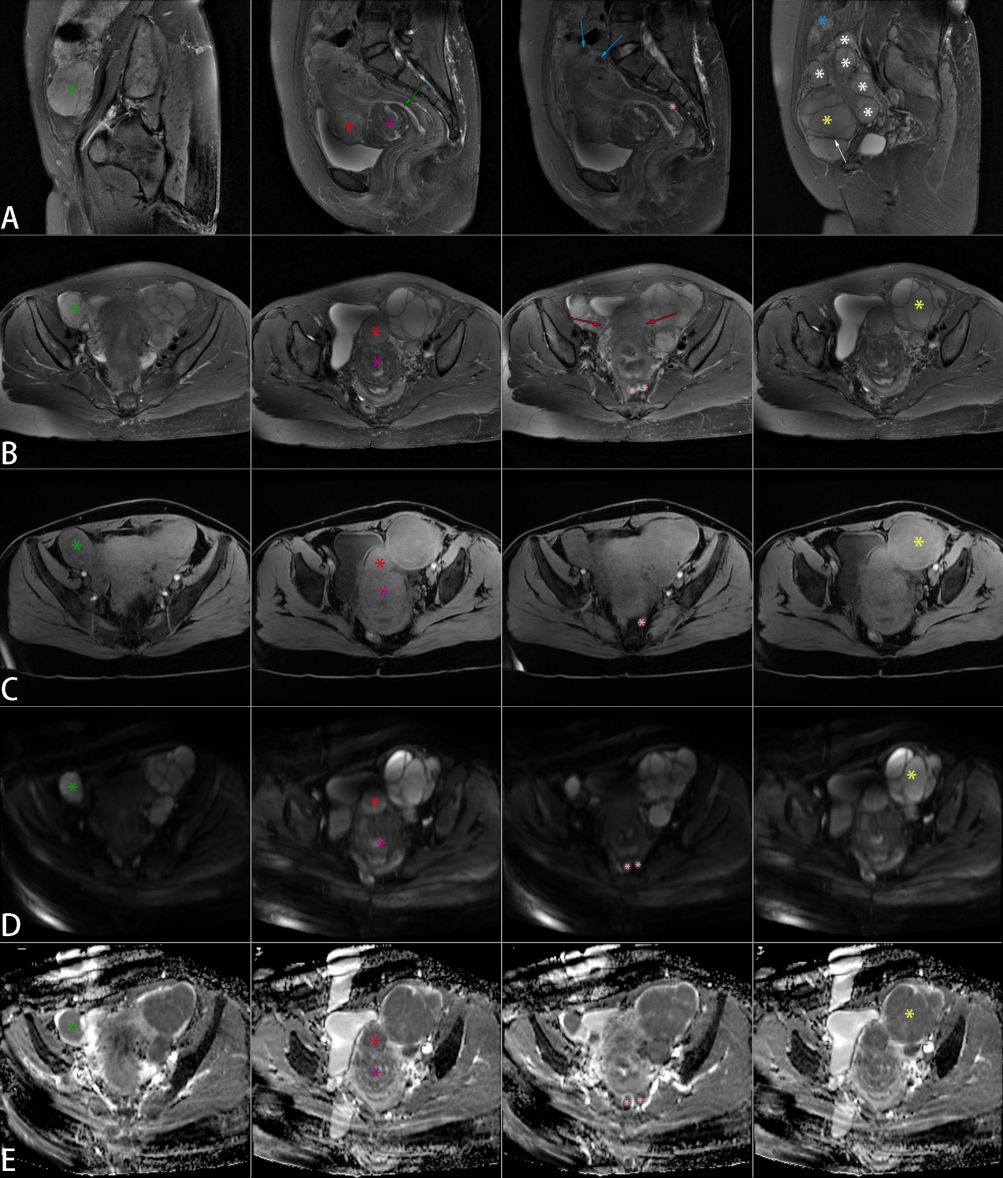
Non-contrast pelvic MRI. Multiple space-occupying lesions were present in the uterus and pelvis, with significant masses marked on sagittal T2WI **(A)**, axial T2WI **(B)**, axial T1WI **(C)**, axial DWI **(D)**, and axial ADC **(E)** images. The uterus displayed irregular enlargement, featuring multiple nodules and mass-like lesions in the myometrium and beneath the serosal layer. Two larger masses were located on the anterior wall. A mass near the cervix (purple star) was isointense with focal mild hypointensity on T1WI and demonstrated mixed signal on T2WI, with patchy hyperintense foci within a hypointense background. Another mass (red star) was also isointense on T1WI but showed slightly higher signal intensity than the myometrium on T2WI, with uniform density and indistinct borders from the myometrium. Additionally, two smaller round lesions (blue arrows) were present, isointense on T1WI and hypointense on T2WI. Despite the compression of the endometrial cavity, the continuous endometrial signal remained visible (green arrow). Multiple well-defined lesions were observed in the bilateral adnexa, isointense on T1WI and hyperintense on T2WI. The left lesion (yellow star) was larger, with T2WI hypointense bands (white arrow) separating it, and multiple smaller lesions (white stars) surrounding it. A mass-like lesion (green star) was seen in the right pelvis but appeared slightly hypointense on T1WI. Lesions on both sides of the pelvis merged with the uterus, showing indistinct borders (red arrows). Additionally, multiple nodules were noted in the rectouterine pouch (pink star) and the abdominal wall (blue star), isointense on T1WI and hyperintense on T2WI. The marked tumors on DWI exhibited varying degrees of high signal intensity, with correspondingly low signals on ADC. MRI, magnetic resonance imaging; T2WI, T2-weighted imaging; T1WI, T1-weighted imaging; DWI, diffusion-weighted imaging; ADC, apparent diffusion coefficient.

In July 2024, a 44-year-old woman presented to our hospital with a contrast-enhanced magnetic resonance imaging (MRI) report from six months earlier, which showed “multiple uterine leiomyomas with degeneration, largest diameter 66mm”. Aside from occasional severe dysmenorrhea, she experienced no other discomfort. After conducting a thorough history, we determined that the patient had a history of multiple leiomyomas spanning over ten years. Five years ago, she underwent a laparoscopic myomectomy that successfully excised three tumors, the largest measuring 70mm in diameter using a power morcellator. And the postoperative histopathology confirmed that all the tumors were benign leiomyomas. Postoperatively, the patient attended routine follow-up consultations, and imaging showed a progressive enlargement of the unresected lesions. About a year ago, the largest uterine mass had increased to 50mm in diameter. At that time, the patient underwent transcatheter arterial embolization (TAE) for hepatic hemangioma, along with uterine artery embolization (UAE). Unfortunately, a follow-up examination seven months later revealed that the lesions had not only increased in size and number but also degenerated, leading to her current consultation. In addition, the patient experienced menarche at age 13, had a BMI of 25.39 kg/m², her father succumbed to liver cancer, and she reported no history of long-term exogenous estrogen use. The timeline provides a summary of the patient’s intricate medical journey (***Figure 1 in the appendix***).

**Figure 2:**
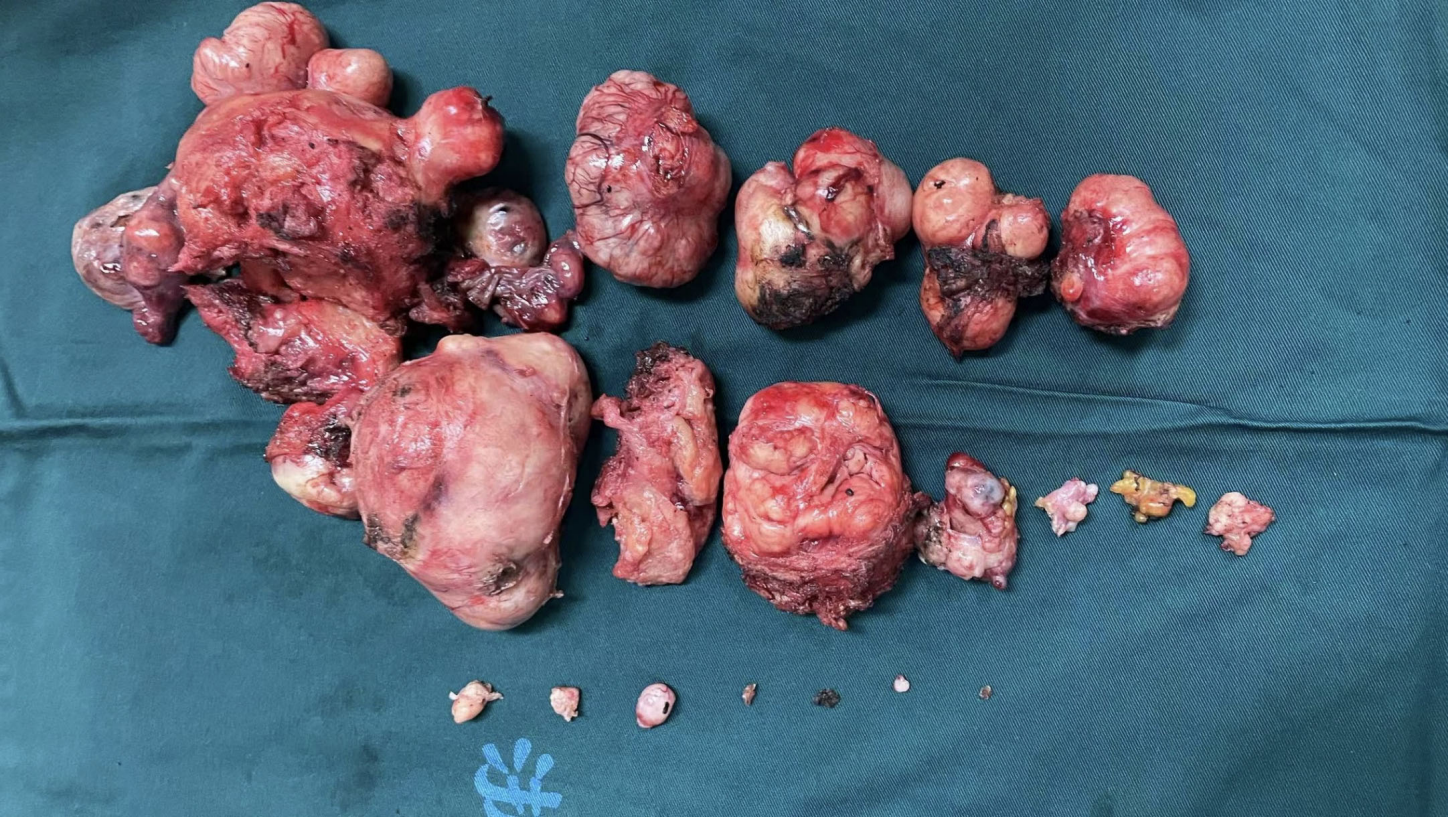
Gross specimen. Gross specimen of the uterus, bilateral adnexa, and pelvic masses.

**Figure 3:**
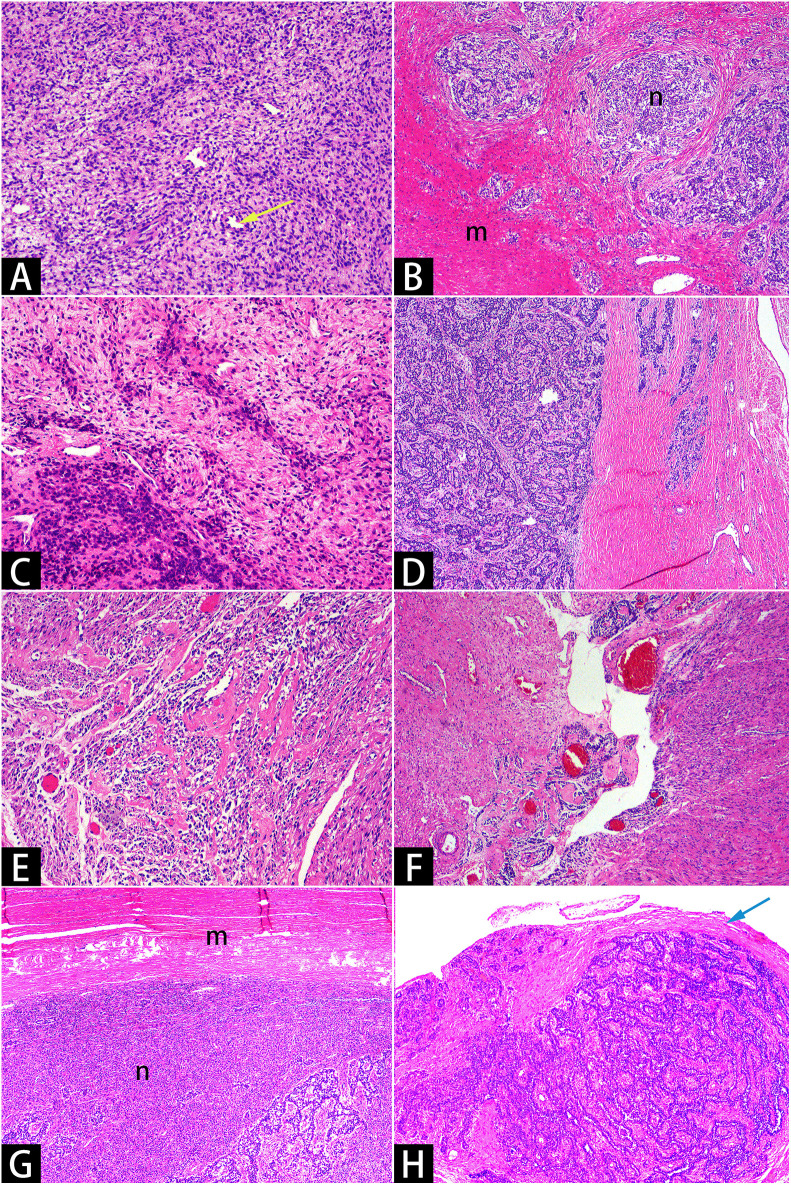
Hematoxylin and eosin staining. Microscopy showed that short, spindle-shaped cells with minimal atypia, resembling proliferative-phase endometrial stromal cells [**(A)**×10]. Tumor cells infiltrated the myometrium in a tongue-like manner [**(B)**×4] and encircled small vessels resembling spiral arterioles in a spiral pattern [**(A)**×10, yellow arrow]. The tumor cells exhibited uneven density [**(C)**x10], with some areas differentiating into sex cord-like [**(D)**×4, **(H)**×4] and smooth muscle-like [**(E)**×10, **(F)**×4] structures. Smooth muscle bundles encircled the uterine masses [**(G)**x4, with varying staining intensities due to uneven section thickness], and reactive hyperplastic collagen fibers were present around the pelvic nodules [**(H)**x4, marked with a blue arrow]. The letter “m” denotes smooth muscle bundles, while the letter “n” denotes sarcomas.

During the physical exam, multiple soft masses were found in the patient’s pelvic cavity, without tenderness, and their connection to the uterus and adnexa was unclear. Subsequently, she received a battery of tests in the hospital. In the tumor marker test, all but serum cancer antigen 125 (CA125), which was slightly higher (45U/ml), were within normal ranges. Ultrasound examination revealed pelvic masses with features suggestive of uterine leiomyomas (***Figure 2 in the appendix***). The plain pelvic MRI revealed “multiple uterine leiomyomas” and “multilocular cystic lesions in bilateral adnexa, with the largest located on the left side” (**Figure 1**). We then performed a contrast-enhanced abdominal computed tomography (CT) to further clarify the diagnosis. The CT images demonstrated features consistent with multiple leiomyomas, with certain masses extending from the uterine contour, ruling out ovarian cysts (***Figure 3 in the appendix***). Additionally, the patient reported that a recent gastrointestinal endoscopy had yielded normal results.

Despite our concerns about the patient’s condition, the intraoperative findings were nonetheless striking. Masses or nodules of varying sizes, with well-defined borders, were observed on the uterus, rectum, peritoneum, mesentery, and ligaments, as well as the vesicouterine pouch and Douglas pouch, during laparoscopic exploration (***Video 1 in the appendix***). We removed some of the tumors from the uterine and round ligament surfaces for an intraoperative frozen section. The results showed “suspected ESS”, which would be confirmed by routine pathology and immunohistochemistry. The surgical method was decided after informing the patient’s family and discussing it together. We separated the uterus and bilateral adnexa, then removed them through the vagina. Additionally, we completely resected and removed the masses in the pelvic peritoneum, mesentery, rectal surface, and vaginal stump. The dissection of the uterus revealed several spherical, leiomyoma-like projections inside the uterine body, with no abnormalities in the uterine cavity (**Figure 2**).

**Figure 4:**
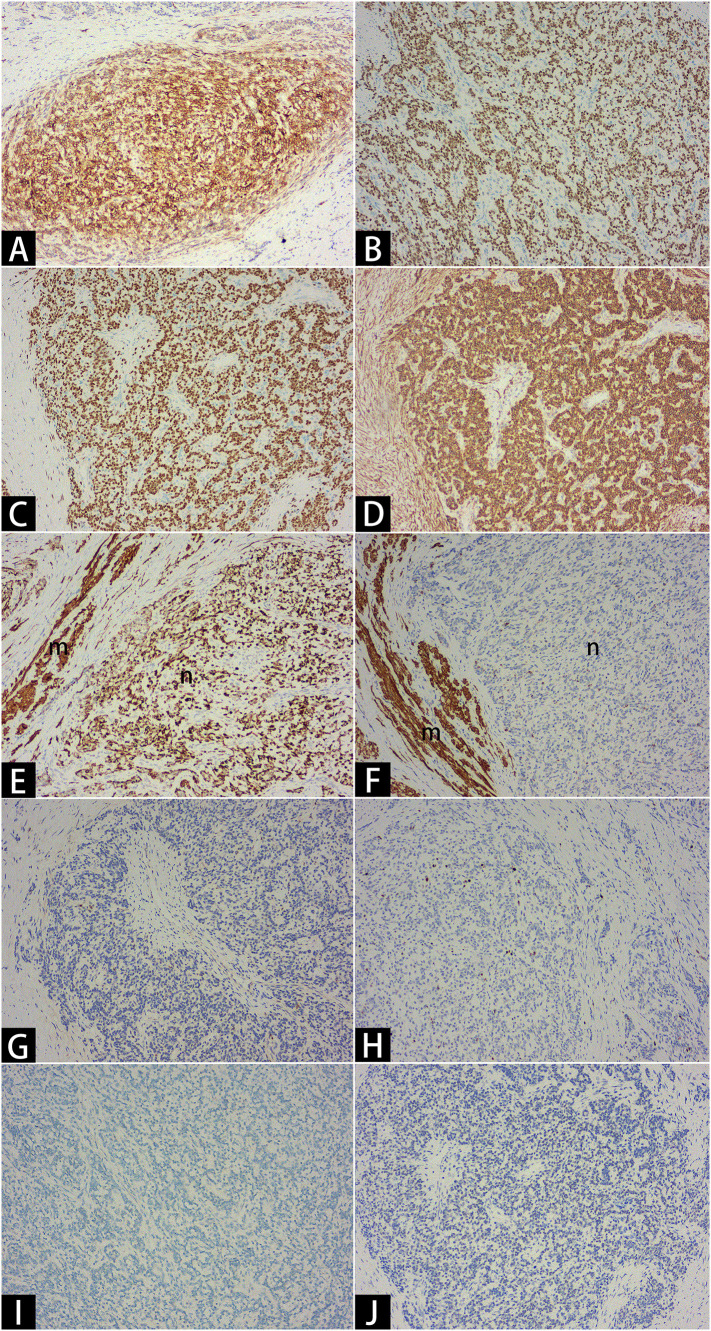
Immunohistochemistry. Critical results: CD10 ++ [**(A)**×10], ER +++ [**(B)**×10], PR +++ [**(C)**×10], α-SMA +++ [**(D)**×10], Desmin +++ [**(E)**×10], Caldesmon - [**(F)**×10], Cyclin D1 - [**(G)**×10], KI67 3% [**(H)**×10], SF1 - [**(I)**×10], WT1 - [**(J)**×10]. The letter “m” denotes smooth muscle bundles, and the letter “n” denotes sarcomas.

Histopathological evaluation, supported by immunohistochemistry, is consistent with a diagnosis of LG-ESS, characterized by sex cord-like and smooth muscle differentiation (**Figures 3**, **4**). Immunohistochemical analysis showed the following staining patterns (***Table 1 in the appendix***): Ki-67 (3%), CD10 (++), ER (+++), PR (+++), etc. The pathologist also provided additional pathological details (***Figure 4 in the appendix***).

The FIGO (2009) staging system (10) classified the patient as stage IIIB owing to tumor invasion of the abdominal tissues, including the mesocolon and parietal peritoneum. Given the patient’s clinical condition (positive ER/PR expression, negative surgical margins, no pelvic lymph node enlargement, and the presence of breast nodules), we selected letrozole (an aromatase inhibitor) to prevent tumor recurrence, in accordance with relevant guidelines and literature. We recommended follow-up every three months for the first two years postoperatively, followed by every six months thereafter, with lifelong follow-up. The results of the three-month postoperative follow-up showed that everything is in order.

## Discussion

This case suggests that morcellation and UAE may accelerate LG-ESS progression, delay diagnosis and treatment, and result in a poor prognosis. Laparoscopic power morcellation is associated with the spread of endometriosis and leiomyomas, with an incidental sarcoma incidence ranging from 0.03% to 1% (11). Given the medical history, the morcellator used during her myomectomy five years ago may have contributed to the spread and implantation of tumor cells, leading to her advanced-stage diagnosis. Besides, upon reviewing the relevant literature, we found a case report (12) describing the rapid enlargement of LG-ESS lesions following UAE, similar to the case presented here. UAE commonly treats uterine benign diseases without a definitive pathological diagnosis, potentially delaying the treatment of undiagnosed uterine malignancies (13). The ischemic-hypoxic microenvironment induced by UAE may stimulate tumor angiogenesis, accelerating growth and promoting metastasis. Furthermore, UAE may compromise ovarian perfusion, causing fluctuations in sex hormone levels. Given the estrogen/progesterone sensitivity of LG-ESS, alterations in these hormones could potentially accelerate tumor progression. Consequently, persistent or enlarging uterine lesions post-UAE warrant immediate investigation to exclude malignancy.

Imaging did not reveal distinct features to reliably identify LG-ESS, contributing to the misdiagnosis (14). In this case, MRI showed the LG-ESS lesions as isointense or slightly hypointense on T1-weighted imaging (T1WI) and exhibited a homogeneous high signal on T2-weighted imaging (T2WI) with internal hypointense bands. The purple-star-marked lesion in **Figure 1** demonstrated MRI features suggestive of a leiomyoma with focal necrosis. However, histopathological examination revealed it to be a leiomyoma harboring LG-ESS. Infiltration of sarcoma cells into the smooth muscle layer resulted in uneven cellular density, producing a mixed-signal mass on T2WI. DWI hyperintensity combined with low ADC values indicates high cellular density, enabling differentiation of uterine sarcomas from ordinary and degenerative leiomyomas but not cellular leiomyomas (15, 16). Placement of 25mm^2^ regions of interest within DWI-hyperintense regions demonstrated significantly lower ADC values (×10^-3^mm²/s) in the LG-ESS lesion compared with normal myometrium (1.28 ± 0.77 vs. 1.63 ± 0.95). While DWI and ADC values demonstrate critical utility in diagnosing LG-ESS, their integration with T2WI features, particularly the hypointense bands within hyperintense lesions, remains essential for differentiation from cellular leiomyomas and other uterine sarcomas (17). Additionally, this case revealed an exceptionally rare LG-ESS-associated leiomyoma exhibiting MRI features indistinguishable from a degenerated counterpart, heightening diagnostic challenge.

Although LG-ESS typically originates in the uterus, it can also arise in various other organs, including the ovaries, peritoneum, bowel wall, vagina, bladder, lungs, etc. The origin of LG-ESS in these non-uterine sites is extremely rare and is often associated with the malignant transformation of endometriotic lesions (18–20). In this case, pathology confirmed a diagnosis of LG-ESS and adenomyosis, with no evidence of tumor cell invasion into the endometrium. The possibility that the tumors originated from ectopic endometrial tissue, specifically adenomyosis, cannot be excluded. However, the direct evidence was not obtained in the pathological sections.

The histological features and invasion pattern distinguish LG-ESS from other uterine tumors (18). Although histological differentiation may vary, immunohistochemistry provides further clarity for a more accurate diagnosis (21). Most LG-ESS cases show chromosomal rearrangements, particularly the t(7;17)(p15;q21) translocation, which causes JAZF1-SUZ12 gene fusion. However, molecular testing is not routine but is used when the onset site is atypical or tissue differentiation presents difficulties (22, 23).

The preferred treatment for LG-ESS is total hysterectomy (without morcellation) combined with bilateral salpingo-oophorectomy (24), while efforts should also be made to perform tumor debulking for extra-uterine lesions (25). The issues of lymph node dissection and ovarian preservation remain controversial. Some studies suggested that the removal of normal lymph nodes does not significantly affect patient survival (22), and the preservation of normal ovaries does not have a significant relationship with recurrence (26). Theoretically, abdominal surgery should be the preferred approach for patients with suspected or confirmed LG-ESS to reduce recurrence risk (27). We believe that regardless of surgical approach (open or minimally invasive), the key principle is to avoid direct morcellation. The literature reported that transvaginal morcellation of the uterus within a retrieval bag during laparoscopic surgery achieved satisfactory outcomes in treating LG-ESS (28). Currently, systematic evidence regarding the safety of laparoscopic surgery for early-stage LG-ESS remains lacking. In this case, after thorough informed consent, the patient’s family insisted on minimally invasive surgery. Fortunately, owing to favorable pelvic anatomy, the entire uterus and bilateral adnexa were successfully extracted intact transvaginally, avoiding morcellation in the abdominal cavity or vagina.

Given the moderate to high expression of ER and PR in the lesions, adjuvant anti-estrogen therapy (aromatase inhibitors or progestins) is recommended for patients with stage II-IV LG-ESS after surgery to reduce the risk of recurrence (8). The efficacy of radiotherapy and chemotherapy for LG-ESS remains unclear.

## Conclusion

This case carries significant implications for clinicians. The rapid enlargement of uterine masses should raise concern, as imaging could not accurately differentiate LG-ESS. Additionally, treatments for benign uterine conditions, such as the application of morcellation and UAE, may promote the development of potential LG-ESS. We speculate that endometriotic lesions within the myometrium may be another potential origin of LG-ESS. If this hypothesis is confirmed, then actively treating the primary disease, exercising caution with morcellation, and maintaining regular follow-ups may be the most effective preventive strategy available. Surgery is the preferred treatment, with aromatase inhibitors or progestins helping to prevent recurrence. While this article offers insights into occult malignancies, its conclusions are limited as a case report and require further investigation.

## Data Availability

The original contributions presented in the study are included in the article/**Supplementary Material**. Further inquiries can be directed to the corresponding author.
