# Supplementary material for: Low-grade endometrial stromal sarcoma: a case report and literature review
Source: Front Oncol. 2025 Aug 25;15:1652010. doi: 10.3389/fonc.2025.1652010 (PMC12414757; doi:10.3389/fonc.2025.1652010)
Supplement: Supplementary file 1 [file DataSheet1.docx]

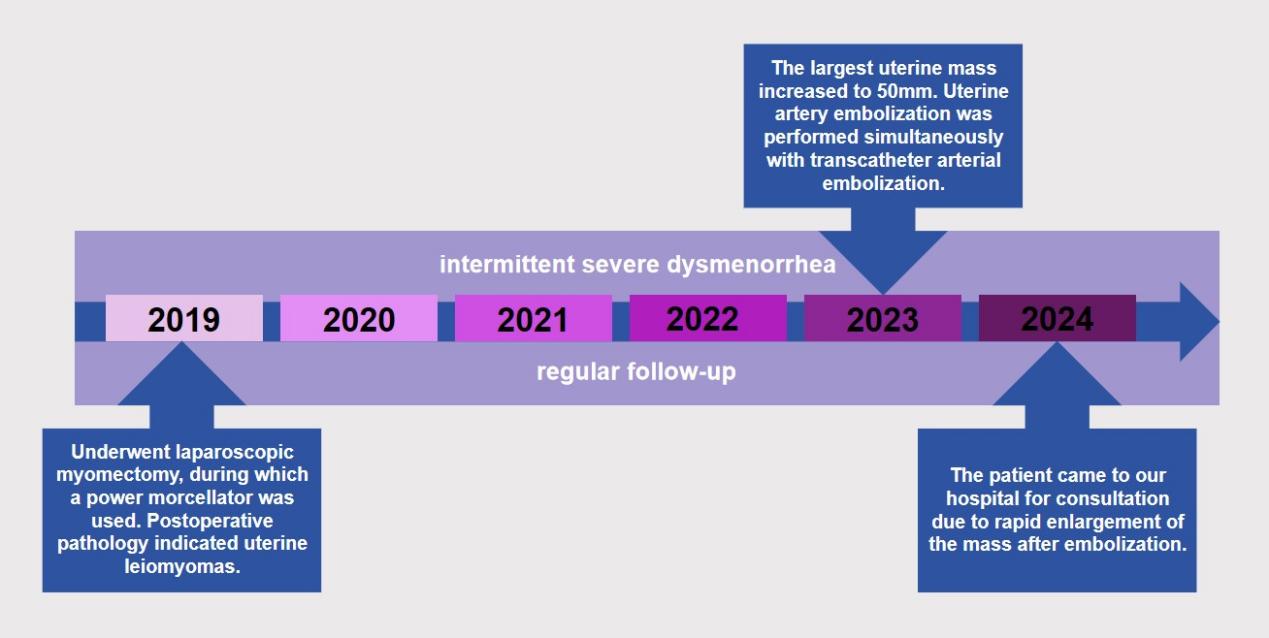


**Figure 1** Timeline of the Disease Course

**
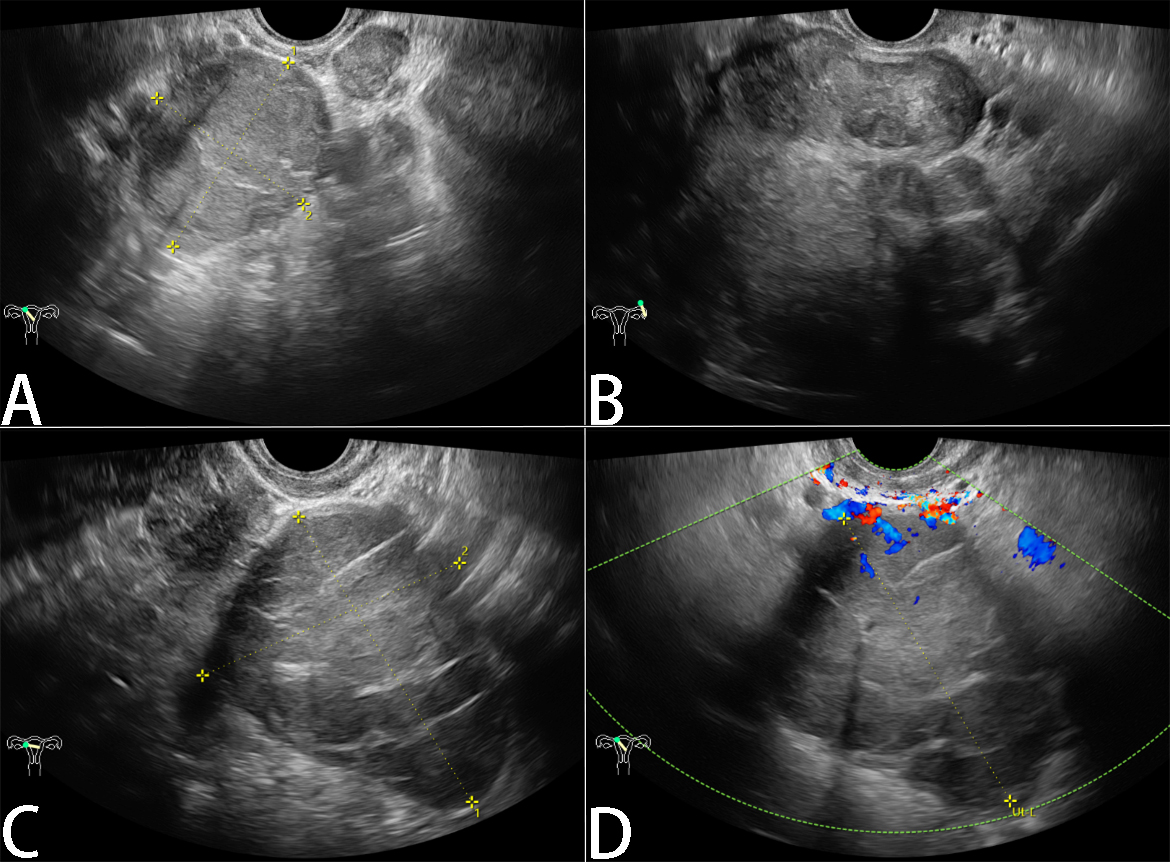
**

**Figure 2** Transvaginal Ultrasonography

The myometrium showed heterogeneous echogenicity with multiple round or oval hypoechoic nodules, some protruding toward the serosa. The largest nodule, on the left anterior wall, had a clear boundary and homogeneous echogenicity. Color Doppler imaging showed blood flow signals.


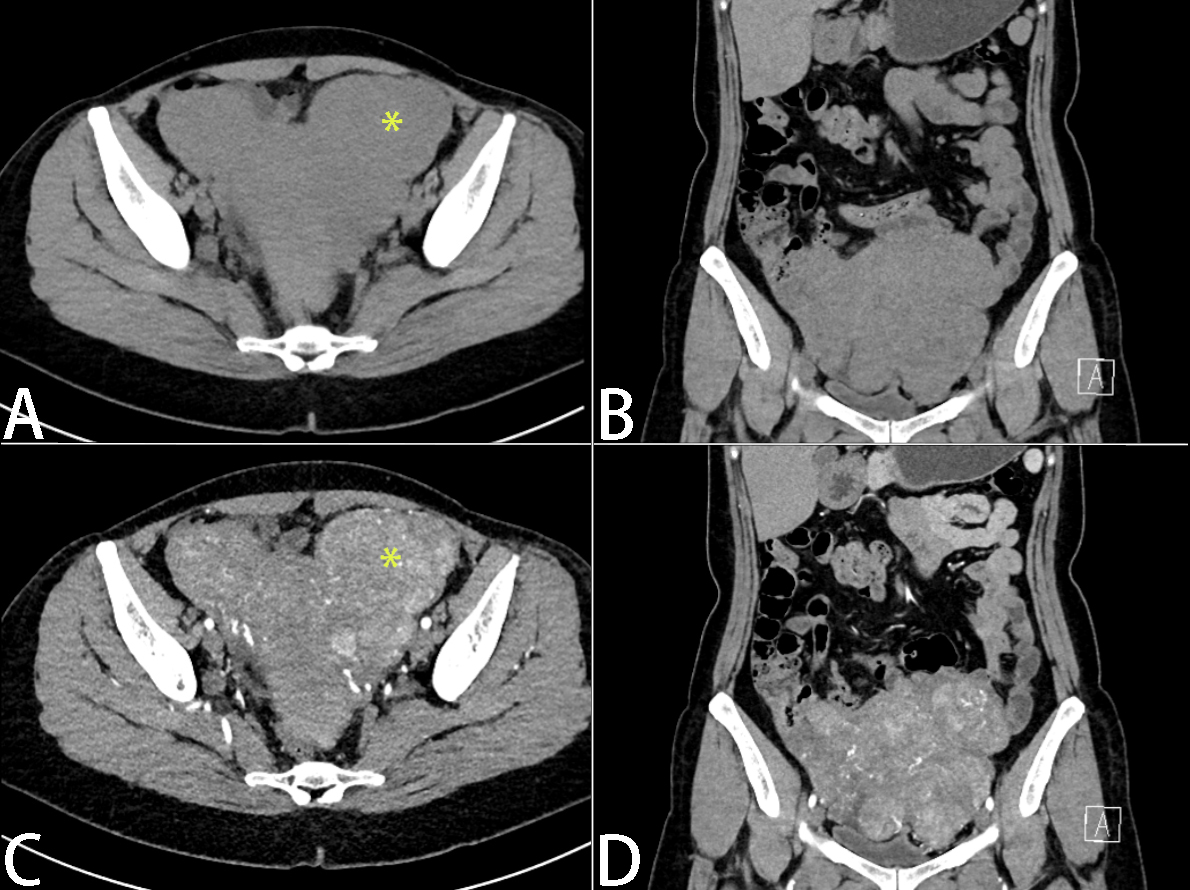


**Figure 3** Contrast-enhanced Abdominal CT

The plain abdominal CT images (A, B) showed multiple isodense masses in the pelvis, without clear demarcation from the uterus, the largest of which was located in the left pelvis (yellow star). In contrast-enhanced scans (C, D), the masses exhibited marked enhancement. The pelvis, abdomen, and retroperitoneum showed no enlarged lymph nodes.

Note: CT: computed tomography.

**Video 1** Laparoscopic Exploration

The omentum was attached to the uterine surface, with prominent dilated blood vessels. The uterus was enlarged, approximately the size of a 3-month pregnancy, with an uneven surface, over 20 myoma-like protrusions with a soft texture, and a dense network of blood vessels on the tumour surfaces. The largest tumour, approximately 7×6 cm, was located on the anterior left wall of the uterus, firmly adherent to the peritoneum. A myoma-like protrusion measuring 5×5 cm was seen at the cervix. Other lesions ranged in size from 0.5 to 4 cm. The appearance of the bilateral fallopian tubes and ovaries was normal. Several hard, round lesions, approximately 0.5-1 cm in size, were visible on the surface of the pelvic peritoneum, with clear borders from the surrounding tissues. An irregular, firm mass, approximately 3.5×2 cm, was observed in the left mesocolon. Multiple nodules, ranging from 0.5 to 2 cm, were present on the left uterosacral ligament, right ovarian suspensory ligament, round ligament of the uterus, vesicouterine pouch, Douglas pouch, rectal surface, and the anterior right peritoneal wall. The omentum, diaphragm, liver, spleen, stomach, and pelvic lymph nodes showed no significant abnormalities.

| **Outcomes** | **Markers** | | | | | | |
| --- | --- | --- | --- | --- | --- | --- | --- |
| Positive | CD10 | ER | PR | alpha-SMA | Desmin | Vimentin | CD56 |
| Negative | CD34 | CD117 | Cyclin D1 | Caldesmon | EMA | SF1 | WT1 |
| Others | There were no changes to p53, and the mitotic rate, as measured by Ki-67, was only 3%. | | | | | | |

**Table 1** Immunohistochemical Results

**
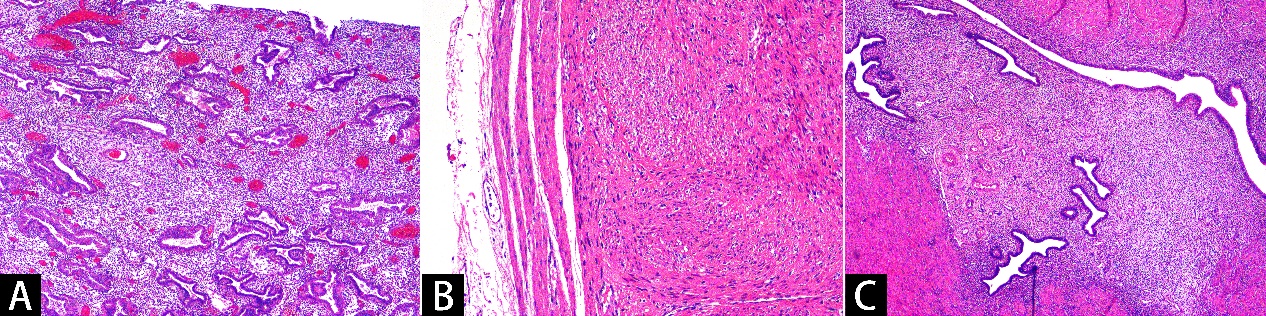
**

**Figure 4** Hematoxylin and Eosin Staining

No tumour cell involvement in the endometrium, showing a normal secretory phase (A×4); leiomyomas (B×10) and ectopic endometrial lesions (C×4) in the myometrium.
